# MiRAR—miRNA Activity Reporter for Living Cells

**DOI:** 10.3390/genes9060305

**Published:** 2018-06-19

**Authors:** Matthew A. Turk, Christina Z. Chung, Emad Manni, Stephanie A. Zukowski, Anish Engineer, Yasaman Badakhshi, Yumin Bi, Ilka U. Heinemann

**Affiliations:** 1 Department of Biochemistry, The University of Western Ontario, 1151 Richmond Street, London, ON N6A 5C1, Canada; mturk5@uwo.ca (M.A.T.); cchung88@uwo.ca (C.Z.C.); emanni@uwo.ca (E.M.); szukowsk@uwo.ca (S.A.Z.); ybadakhs@uwo.ca (Y.B.); ybi@uwo.ca (Y.B.); 2 Department of Physiology and Pharmacology, The University of Western Ontario, 1151 Richmond Street, London, ON N6A 5C1, Canada; aengine@uwo.ca

**Keywords:** fluorescent reporter, live cell imaging, microRNA quantification, optogenetics, small molecule drug screening

## Abstract

microRNA (miRNA) activity and regulation are of increasing interest as new therapeutic targets. Traditional approaches to assess miRNA levels in cells rely on RNA sequencing or quantitative PCR. While useful, these approaches are based on RNA extraction and cannot be applied in real-time to observe miRNA activity with single-cell resolution. We developed a green fluorescence protein (GFP)-based reporter system that allows for a direct, real-time readout of changes in miRNA activity in live cells. The miRNA activity reporter (MiRAR) consists of GFP fused to a 3′ untranslated region containing specific miRNA binding sites, resulting in miRNA activity-dependent GFP expression. Using qPCR, we verified the inverse relationship of GFP fluorescence and miRNA levels. We demonstrated that this novel optogenetic reporter system quantifies cellular levels of the tumor suppressor miRNA let-7 in real-time in single Human embryonic kidney 293 (HEK 293) cells. Our data shows that the MiRAR can be applied to detect changes in miRNA levels upon disruption of miRNA degradation pathways. We further show that the reporter could be adapted to monitor another disease-relevant miRNA, miR-122. With trivial modifications, this approach could be applied across the miRNome for quantification of many specific miRNA in cell cultures, tissues, or transgenic animal models.

## 1. Introduction

Human gene expression and RNA transcript stability can be regulated before, during, and after transcription. MicroRNAs (miRNAs) regulate transcript stability by binding to messenger RNAs (mRNAs) in complementary regions, inducing endonuclease-mediated cleavage or inhibiting protein synthesis [1]. In mammals, miRNAs usually contain sequence homology to their target transcript in their 5′ and 3′ untranslated regions (UTR) [2,3]. Genes encoding miRNAs are transcribed as long precursor transcripts and processed to yield short 18–24-nucleotide-long miRNAs. These miRNAs are subsequently integrated into protein complexes to induce mRNA silencing. While translational aspects of miRNA research are primarily focused on cancer, deregulation of miRNA stability and activity has relevance to many diseases. Dysfunctional miRNA expression, processing, and degradation have been found in diseases including breast cancer [4], acute myeloid leukemia [5], ovarian cancer [6], and hepatocellular carcinoma [7], but links between miRNAs and Alzheimer’s disease [8], diabetes [9], and schizophrenia [10] are also emerging.

The miRNA let-7 is often implicated in disease and the let-7 miRNA sequence and timing of expression during development are highly conserved amongst vertebrates [11]. In normal cells and tissues, let-7 suppresses tumor proliferation and cell survival by negatively regulating oncogenic signaling pathways [12]. Let-7 directly binds to complementary regions of mRNAs with protein products involved in cell cycle proliferation and apoptosis, such as e.g., Ras, high mobility group A2 (Hmga2), Caspase 3, and others [11,13,14,15,16,17]. Let-7 levels are significantly lower in cancer cells and stem cells compared to differentiated cell types, highlighting the role for let-7 in cell cycle regulation [18,19]. Similarly, let-7 is down-regulated in numerous cancers [20,21,22] and low let-7 levels are associated with shortened post-operative survival [23].

Recent work has begun to reveal the role of let-7 in maintaining cell differentiation and cancer proliferation [12,13,15]. In lung cancers, let-7 and the oncogene Kirsten rat sarcoma viral oncogene homolog (*kras*) have a reciprocal relationship [16]. High KRas levels and low let-7 levels generate a highly cancerous phenotype. Increasing let-7 levels, however, cause KRas levels to decrease and normal cell morphology to return. The KRas mRNA has seven predicted let-7 binding sites in its 3′-UTR [16]. Other genes regulated by let-7 include *Hmga2* and *Caspase 3*. *Hmga2* regulates the G2/M checkpoint in cell cycling and contains binding sites for let-7 miRNA in its 3′-UTR [24]. Let-7 also regulates apoptosis via let-7 binding sites in the 3′-UTR of *Caspase 3*. By interfering with *Caspase 3* expression, let-7 allows cells to escape apoptotic effector caspases [17].

*KRas*, *Hmga2*, *Caspase 3*, and other oncogenes are directly regulated by let-7 levels in the cell. Thus, let-7 biosynthesis and the regulation of let-7 levels are of increasing interest as new therapeutic targets [25]. Current treatments have focused on a let-7 replacement strategy [20], yet the delivery of RNA therapeutics has proven difficult [26]. Another approach focuses on inhibitors of let-7 degradative enzymes Lin28 [27,28] and the terminal uridylyltransferase Tut4 [29] as targets for small molecule chemotherapeutics. Lin28 binds to precursor miRNA (pre-miRNA) let-7 and recruits Tut4, which subsequently polyuridylates the pre-miRNA. Polyuridylated RNAs are degraded by the U-specific exonuclease Dis3L2 [1]. Screening for small molecule inhibitors of let-7 degradative enzymes currently relies on in vitro biochemical assays to screen for functional inhibition of the respective proteins. Unfortunately, identified small molecule inhibitors often fail to effectively alter miRNA metabolism in vivo due to off-target activities, unspecific side effects, and failure to efficiently enter the cell. Screens directly assessing miRNA levels and activity in the cell would circumvent these technical difficulties. Several methodologies are available to assess overall and specific expression levels of miRNAs in cells. Next-generation sequencing and miRNA arrays are used to identify changes in the overall miRNome. Real-time quantitative polymerase chain reaction (RT-qPCR) and northern blotting are tools to probe individual miRNAs [30,31], yet these assays are not amenable to studies in live cells and they fail to report the level of active miRNA.

The observed variability between tissues and even between single cells call for the development of methods to follow expression and activity of miRNAs in tissues or individual living cells [27,28,29]. Furthermore, miRNA quantity does not necessarily correspond to miRNA activity, as miRNAs can be silenced by single nucleotide additions without affecting miRNA prevalence in the cell [32]. We developed an optogenetic green fluorescence protein (GFP)-based reporter to assess the level of active let-7 in live cells. We fused a let-7 regulated 3′-UTR from the human *kras* gene to a GFP reporter, allowing for a direct readout of let-7 activity in vivo, thus generating a miRNA activity reporter (MiRAR). We further show that this MiRAR reporter system can be adapted as a reporter for other miRNAs. Our proof using principle experiments shows that genetically encoded sensors of miRNA activity will be highly useful tools in investigating the biological role of RNA-regulating enzymes in vivo.

## 2. Materials and Methods

### 2.1. Genomic DNA Extraction

Genomic DNA was prepared as a template for the amplification of 3′-UTRs as described previously [33]. Briefly, human embryonic kidney 293 (HEK 293) cells were grown to confluency in Dulbecco’s modified eagle medium (DMEM) from Gibco (Thermo Fisher Scientific, Waltham, MA, USA) and 10% fetal bovine serum (FBS). Cells from half of a 150 mm plate (~10^7^ cells) were resuspended in 2.4 mL lysis buffer (0.6% sodium dodecyl sulfate (SDS), 10 mM ethylenediaminetetraacetic acid (EDTA), 10 mM Tris-HCl pH 8.0) and 25 units RNase I (NEB M0243S) and incubated at 37 °C for 1 h. This was followed by the addition of 240 µL 5 M NaCl (6 mmol) and 1 h of incubation on ice. The solution was centrifuged at 10,000× *g* for 30 min at 4 °C and the DNA was extracted from the supernatant via phenol chloroform extraction. The extracted DNA was stored at −20 °C until further use.

### 2.2. Reporter Gene Construct for Let-7

We generated a reporter system to link miRNA content in the cell to GFP fluorescence. GFP was cloned into pcDNA3.1. The 3′-UTR of human wildtype *kras* was amplified from pRL KRas 3′-UTR (plasmid 14804, Addgene, Cambridge, MA, USA) and mutant KRas 3′-UTR from pRL KRas 3′-UTR (plasmid 14805 Addgene) [34] using primers KRasfor (5′-TCTGGGTGTTGATGATGCCTTC-3′) and KRasrev (5′-CCTGGTAATGATTTAAATGTAGTTATAGAAATAAATAATATG-3′). The resulting PCR product was cloned downstream of GFP into pcDNA3.1-GFP using *Kpn*I and *Bam*HI restriction sites, yielding the plasmid pMiRAR-let-7 and pMiRAR-let-7-mutant. Successful cloning was verified by DNA sequencing at the London Regional Genomics Centre (London, ON, Canada). The construct sequences are supplied in the supplementary file S1.

### 2.3. Reporter Gene Construct for miR-122

As a reporter system for miR-122, we chose the cytoplasmic polyadenylation element binding protein (CPEB) 3′-UTR. The CPEB 3′-UTR (NM_001079533.1) was amplified from HEK 293 genomic DNA with primers CPEB-*Kpn*I-for (5′-ATCAGGTACCTAAAGGAGCTGGCCTTG-3′) and CPEB-*Bam*HI-rev (5′-TTAAGGATCCCTGCTGCAACGTGTT-3′). The amplified DNA was then inserted into pCDNA3.1 downstream of GFP, yielding pMiRAR-miR-122. Successful cloning was verified by DNA sequencing at the London Regional Genomics Centre. The construct sequence is supplied in the supplementary file S1.

### 2.4. Quantification of Green Fluorescent Protein Fluorescence in Live Cells

HEK 293 cells were grown to ~80% confluency as described above on a 6-well plate using DMEM supplemented with 1% v/v penicillin-streptomycin (Wisent Inc., Saint-Jean-Baptiste, QC, Canada). Cells were then co-transfected with pMiRAR plasmids, pCMV-tdTomato (632534, Clontech, Mountain View, CA, USA), and RNAs as indicated using Lipofectamine 2000 in Opti-MEM transfection media (11668019, Invitrogen, Carlsbad, CA, USA). Cells were harvested 48 h after transfection. Cell fluorescence was measured using the Synergy H1 microplate reader (BioTek, Winooski, VT, USA) at excitations of 480 nm and 554 nm, and emissions at 509 nm and 581 nm. In each well, a grid of 11 × 11 fields was scanned and fluorescence intensity was recorded. The data reported represent the average GFP and tdTomato fluorescence intensity per well. RNAs co-transfected were as follows: let-7 (5′-p-UGAGGUAGUAGGUUGUGUGGUU-3′) and anti-let-7 (5′-p-AACCACACAACCUACUACCUCA-3′) at 80 pM concentration; hsa-miR-122 (5′-p-UGGAGUGUGACAAUGGUGUUUG-3′) and anti-hsa-miR-122 (5′-p-CAAACACCAUUGUCACACUCCA-3′) at 100 nM. Tut4 knockdown was carried out with anti-Tut4 small interfering RNA (siRNA) (Dharmacon OnTargetPlus System, L-021797-01-0005, Lafayette, CO, USA) according to manufacturer’s instructions. Successful Tut4 knockdown was confirmed by separating 50 μg of total protein from HEK 293 cells treated with Tut siRNA or a scrambled control via SDS-PAGE (SDS-polyacrylamide gel electrophoresis). Proteins were transferred to a polyvinylidene difluoride (PVDF) membrane by western blotting and Tut4 and GAPDH were detected with protein specific antibodies 18980-1-AP (Proteintech, Chicago, IL, USA) and MAB374 (Sigma-Aldrich, St. Louis, MO, USA). All experiments were carried out at least in triplicate; representative cell images are shown.

### 2.5. MicroRNA Quantification by Real-Time Quantitative Polymerase Chain Reaction

RT-qPCR was performed as described previously [35]. Briefly, a primer with an internal stem loop structure was designed to target mature let-7 miRNA (5′-GTTGGCTCTGGTGCAGGGTCCGAGGTATTCGCACCAGAGCCAACAACTAT-3′) or miR-122 (5′-GTCGTATGCAGAGCAGGGTCCGAGGTATTCGCACTGCATACGACCAAACA-3′). This primer was unfolded for 5 min at 65 °C and then refolded for 2 min on ice to form a stem loop structure. The primer was then incubated with total purified cellular RNA. Then, 0.125 pmol RNA was synthesized into complementary DNA (cDNA) using SuperScript III RT (200 units/μL) and stem loop primers. cDNA synthesis was carried out for both RNA extracted from wildtype and Tut4-knockdown cell lines. The reaction was incubated in a thermocycler for 30 min at 16 °C, followed by pulsed RT of 60 cycles at 30 °C for 30 s, 42 °C for 30 s, and 50 °C for 1 min. The cDNA generated was later diluted 10-fold, and quantitative PCR was conducted using SYBR Green qPCR MasterMix (Thermo Fisher Scientific) and qPCR primers (300 nM). Forward primers were designed for miR-122 (5′-AGGCTGGAGTGTGACAATG-3′), let-7 (5′-TGAGGTAGTAGGTTGTATAGTTGTTGG-3′) and universal miR reverse (5-GAGCAGGGTCCGAGGT-3′). Samples were amplified for 35 cycles with Eppendorf Realplex (Eppendorf, Hamburg, Germany), and miRNA levels were extrapolated using a comparative C_T_ (Cycle Threshold)method described previously [35].

## 3. Results

### 3.1. Let-7 micro RNA Reduces Green Fluorescent Proteins Fluorescence in Live Cells

**Figure 1:**
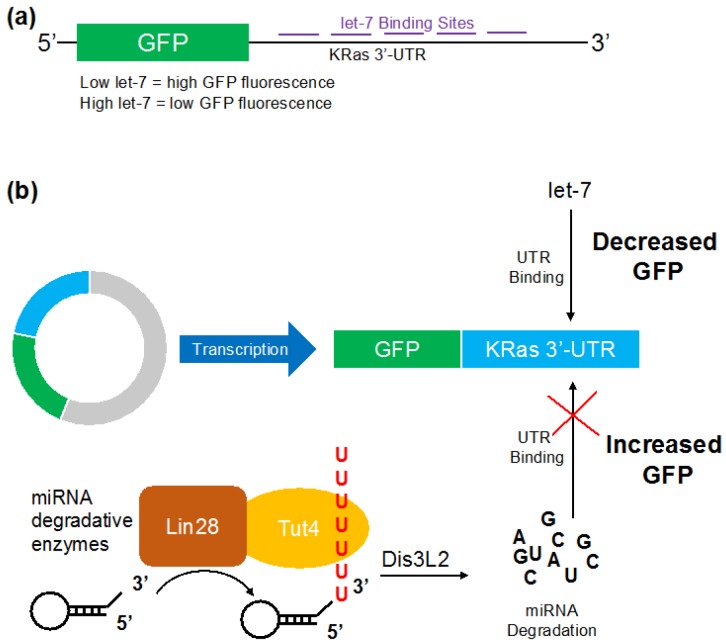
Schematic of the microRNA (miRNA) activity reporter (MiRAR) for let-7 levels in vivo. The KRas 3′-UTR was fused downstream of green fluorescence protein (GFP) to allow quantification of cellular let-7 levels. (**a**) Schematic of pMiRAR-let-7 construct; (**b**) miRNA degradative enzymes Lin28 and Tut4 collaborate to mark let-7 miRNA for degradation by the exonuclease Dis3L2. The RNA binding protein Lin28 recruits Tut4 to polyuridylate miRNA and pre-miRNAs, leading to degradation by the U-specific exonuclease Dis3L2. Lowered miRNA levels lead to an increase in GFP translation and fluorescence. KRas: Kirsten rat sarcoma viral oncogene homolog, UTR: untranslated region.

**Figure 2:**
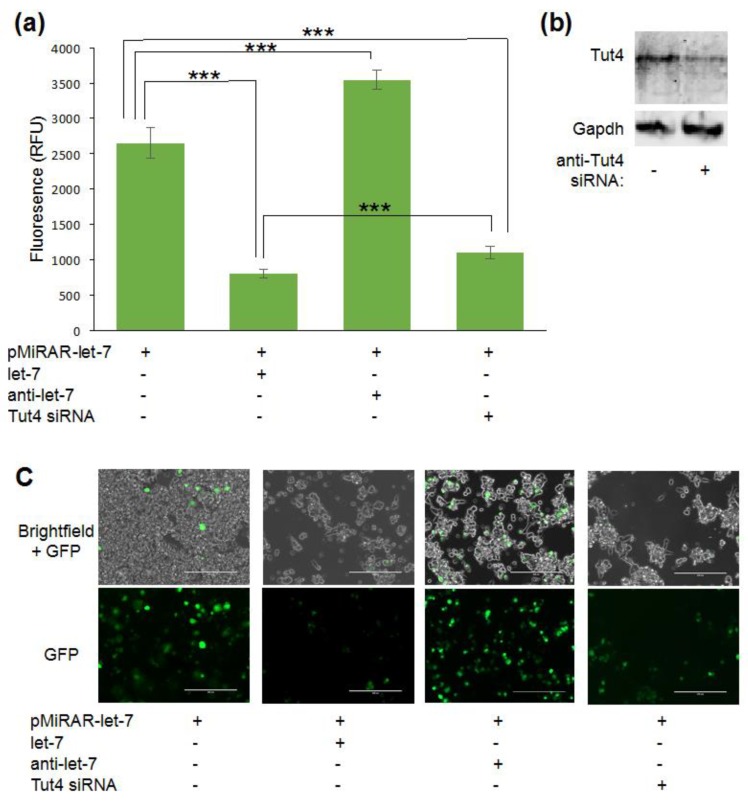
The MiRAR in live cells. Fluorescence intensity measurements and cell images for different treatments of MiRAR-transfected cells. Human embryonic kidney 293 (HEK 293) cells were grown to confluency, transfected with the MiRAR containing the KRas-3′-UTR, and treated as outlined. (**a**) Fluorescence intensities validate miRAR-let-7 as a miRNA reporter. Background fluorescence of untreated cells was subtracted from the experiments. Error bars are based on at least three biological replicates and represent one standard deviation; (**b**) Western blot of untreated HEK 293 cells and treated cells after a knockdown of Tut4, confirming partial depletion of Tut4; (**c**) Images of live cells co-transfected with MiRAR and indicated as small interfering RNAs (siRNAs) or miRNAs. Row 1: overlay of phase light microscopy and GFP UV microscopy; row 2: GFP UV microscopy alone. The white bar represents 200 µm. *p* values are *** < 0.001.

To assess miRNA levels in live cells, we generated a GFP-based reporter system. The 3′-UTR of KRas was cloned downstream of a *gfp* gene into pcDNA3.1 to generate a reporter system where GFP fluorescence is responsive to changes in let-7 concentration in the cell (Figure 1a). Cells transfected with the reporter construct pMiRAR-let-7 expressed GFP (Figure 2a,c), indicating that endogenous let-7 levels do not entirely silence *gfp* expression. Background fluorescence of cells without miRAR (1858 ± 44 RFU (relative fluorescence units)) was subtracted from the fluorescence intensities. To evaluate the responsiveness of GFP production, we co-transfected the reporter pMiRAR-let-7 with 80 pMlet-7 miRNA or separately with anti-miR RNA complementary to let-7 (anti-let-7). Supplementing cells with exogeneous let-7 effectively inhibited GFP translation, reducing fluorescence by more than 3-fold (Figure 2a,c). In contrast, supplementing cells with anti-let-7, which binds to and de-activates cellular let-7, led to a marked decrease in active let-7 molecules in the cell as reported by a 1.3-fold increase in GFP production and fluorescence (Figure 2a,c).

### 3.2. Visualizing Let-7 Accumulation due to Inhibition of Let-7 Degradative Enzymes

We further tested the reporter system by assessing miRNA levels in cells depleted for the let-7 degradative enzyme Tut4, which has been shown previously to affect miRNA degradation [1,29,36,37]. Tut4 polyuridylates let-7 miRNAs, marking them for degradation by the exonuclease Dis3L2 (Figure 1b) [1]. Tut4 was knocked down using siRNA, and partial knockdown of ~50% of Tut4 was confirmed by western blotting (Figure 2b). As expected, the depletion of Tut4 resulted in a decrease of GFP fluorescence by 2.4-fold, confirming an increase in cellular let-7 levels (Figure 2a,c). Thus, elevation of miRNA concentrations caused by inhibition of the uridylyltransferase Tut4, and the subsequent lack of U-dependent let-7 degradation can be measured using our GFP reporter system.

### 3.3. Mutation of Let-7 Binding Sites Abolishes the Sensitivity of the pMiRAR to Changes in Let-7 Levels

**Figure 3:**
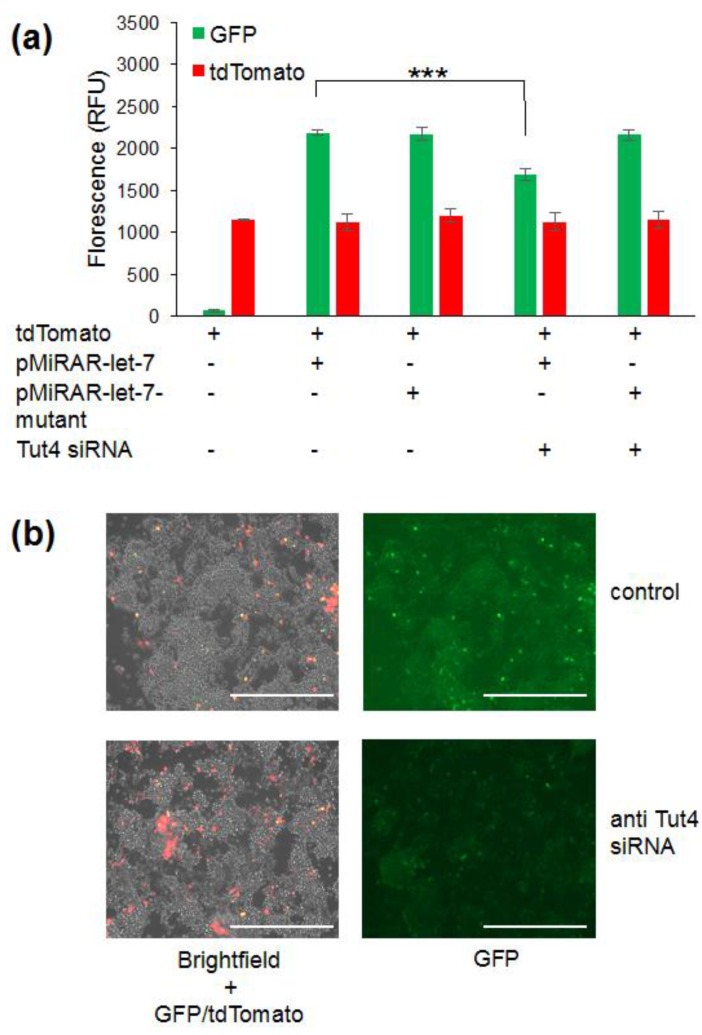
MiRAR–let-7 with KRas-3-UTR is let-7 specific. Fluorescence intensities (**a**) and cell images (**b**) for HEK 293 cells co-transfected with pMiRAR-let-7 and tdTomato. HEK 293 cells were grown to confluence and treated as outlined. Cells were either transfected with the original pMiRAR-let-7 construct (Wild Type (WT)-KRas) or a construct containing mutated let-7 binding sites (pMiRAR-let-7-mutant). Error bars are based on three biological replicates and show one standard deviation. White bars represent 200 µm. *p* values are *** < 0.001.

**Figure 4:**
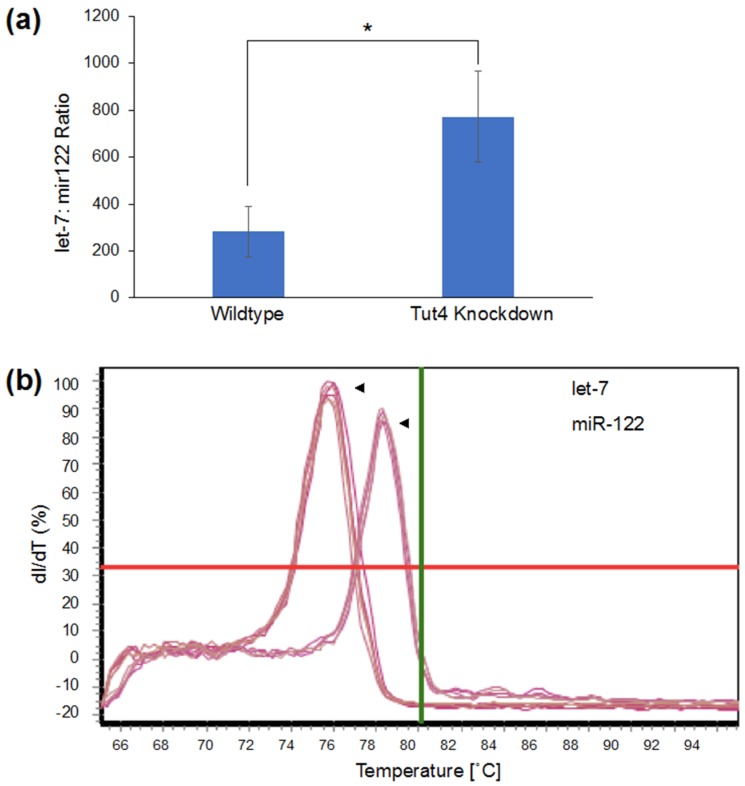
qPCR for let-7 miRNA in wildtype and Tut4-knockdown HEK293 cells. Cells were grown to confluence and total RNA was extracted. (**a**) Let-7 levels were quantified in relation to miRNA miR-122. miRNA levels in the wildtype and Tut4 knockdown are significantly different (*p* < 0.03). Error bars are based on three biological replicates and show one standard deviation. (**b**) Melting curves of let-7 and miR-122 primers are distinct and specific. *p* values are * < 0.05.

To confirm that the changes in fluorescence were indeed exclusively due to let-7 binding to the KRas-UTR in our reporter, we generated a variant of the reporter gene construct with mutated let-7 binding sites. Mutations in the let-7 binding sites abolish the regulatory effect of let-7 on gene expression. The let-7 un-responsive KRas-3′UTR mutant was described previously [34], and cloned downstream of the *gfp* coding sequence. As before, we observed a significant decrease in florescence in cells co-transfected with the wildtype pMiRAR-let-7 construct with Tut4 siRNA compared to untreated cells (Figure 3a,b). In contrast, GFP fluorescence in cells carrying a plasmid with a mutated KRas-UTR fused to GFP (pMiRAR-let-7-mutant) did not respond to a Tut4 knockdown (Figure 3a,b). These data confirm that the change in fluorescence is indeed due to binding of let-7 to the KRas-3′-UTR. As a control experiment, a second plasmid coding for tdTomato was co-transfected to probe for differences in transfection efficiency. No significant changes in transfection efficiency of tdTomato were observed (Figure 3a), indicating that variation in transfection efficiency does not account for the decrease or increase of GFP fluorescence. To further confirm that the Tut4 knockdown decreases let-7 levels in the cell, we quantified let-7 miRNA levels by qPCR (Figure 4). Indeed, in the Tut4 knockdown, a 2.7-fold increase in let-7 miRNA levels was observed, correlating with the 2.4-fold decrease in GFP fluorescence in the pMiRAR-let-7 reporter. These data further confirm that depletion of Tut4 leads to an increase of let-7 levels in the cells, as described previously [29]. We here show that a 2.4-fold decrease in GFP fluorescence corresponds to a 2.7-fold increase in cellular let-7 levels, showing direct inverse reporting of let-7 levels by pMiRAR-let-7.

### 3.4. Adapting the Optogenetic Reporter for Quantifying miR-122

**Figure 5:**
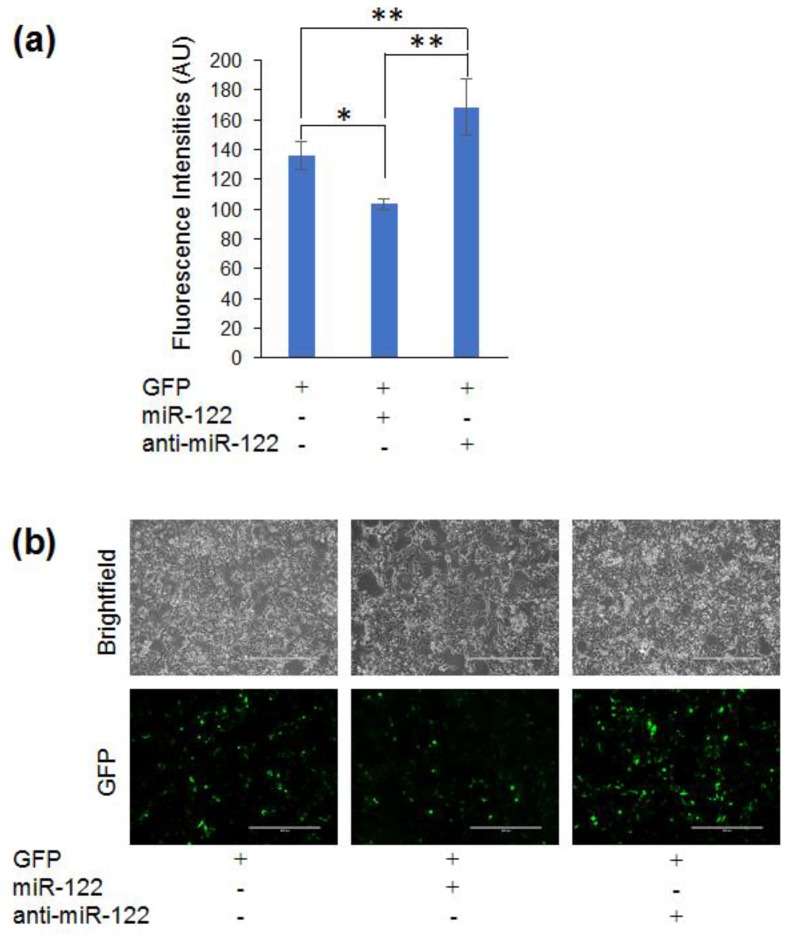
MiRAR-miR-122: Reporter gene construct for cellular miR-122 levels. Fluorescence intensities (**a**) for HEK 293 cells co-transfected MiRAR-miR-122 (CPEB-3′UTR) and miR-122 or anti-miR-122. HEK 293 cells were grown to 60–80% confluency and treated as indicated. Fluorescence intensities were measured by the Synergy H1 microplate reader at an excitation of 480 nm and emission of 509 nm. Error bars represent one standard deviation. (**a**) Cells transfected with 100 nM RNA; (**b**) MiRAR cell images of cells treated with RNAs as indicated. White bars show 400 µm. *p* values are * < 0.05 and ** < 0.01. AU: arbitrary units.

To investigate whether the reporter system is applicable and useful for the reporting of other miRNAs, we generated a similar reporter system for miR-122. The CPEB 3′-UTR encodes 2 miR-122 binding sites and CPEB translation is regulated by cellular miR-122 levels [38]. We fused the CPEB 3′-UTR to *gfp* to generate a reporter system for miR-122 (pMiRAR-miR-122). Low concentrations of co-transfected miR-122 and anti-miR-122 showed no significant change in fluorescence and transfected RNA concentrations were increased to 100 nM. This led to significant changes in GFP fluorescence (Figure 5a). Transfection with miR-122 at 100 nM led to a 1.2-fold decrease in fluorescence, while the anti-miR-122 led to a 1.3-fold increase in fluorescence (Figure 5a,b). Thus, a reporter with two miRNA binding sites is applicable to more pronounced changes in miRNA activity, while a reporter with five miRNA binding sites may be useful for subtle changes. This demonstrates that with minor modification the MiRAR can be adapted to likely any desired target miRNA or range of sensitivity.

## 4. Discussion

The level of miRNA in cells is usually determined by next-generation sequencing or qPCR, which are methods that require cell lysis and subsequent RNA extraction. To observe and quantify miRNA activity in live cells and to capture real-time responses to environmental or chemical changes, a genetically encoded reporter system that allows for a time-resolved quantification of miRNA activity is required. Previously, several luciferase-based reporter systems were generated [34,39,40,41]. These reporters allowed for the quantification of miRNA levels without cell lysis but required the addition of luciferin or a luciferin synthesis plasmid. Luciferase-based reporters provide a snapshot of cellular miRNA levels, rather than the ability to continuously report miRNA activity over an uninterrupted time course. 

To generate a reporter system that is independent of external supplementation with chemicals and allows for a time-resolved quantification of miRNA activity in the cell, we developed a GFP-based miRNA activity reporter. The 3′-UTR of KRas contains several let-7 miRNA binding sites (Figure 1a), and KRas mRNA stability is well known to be regulated by let-7 miRNA [16]. We showed that our reporter accurately reports cellular miRNA concentrations via an inverse response from MiRAR fluorescence. Co-transfected miRNA let-7 or anti-let-7 efficiently reduced or elevated GFP fluorescence in the anticipated inverse relationship. The let-7 reporter displayed high sensitivity, with changes in fluorescence corresponding to pM of miRNA transfected. Thus, we can utilize the reporter to observe small changes in miRNA activity resulting from increasing and decreasing let-7 levels.

Our MiRAR system can also monitor changes in cellular miRNA metabolism. A 2.4-fold decrease in fluorescence corresponds to a 2.7-fold increase in miRNA content upon depletion of the let-7 degradative enzyme Tut4. MiRAR may thus be a highly useful tool to screen small molecule inhibitor libraries for compounds active in altering miRNA metabolism. Previous studies relied on biochemical assays to identify inhibitors of let-7 degradative enzymes Lin28 [27,28] and Tut4 [29]. Utilizing a live-cell reporter to quantify the impact of small molecule inhibitors may lead to a more efficient screening in a physiologically relevant model system. Screening drugs with MiRAR can circumvent time and effort spent on in vitro hits that subsequently fail to permeate the cell or increase let-7 levels in the cell.

For the miRNA let-7, we observed a less pronounced change in GFP fluorescence when adding anti-miR RNA complementary to let-7. This decrease in sensitivity compared to supplementing additional let-7 is most likely due to reaching the level of endogenous let-7 levels in HEK 293 cells. Supplementation of the anti-miRNA will eventually bind and deactivate free cellular let-7, and if provided in excess will not further decrease GFP fluorescence. Thus, the reporter system can also be utilized as a tool to not only quantify changes in let-7 levels, but our data suggest that MiRAR may also be a valuable tool to quantify absolute let-7 levels by titrating increasing concentrations of anti-let-7 until no further increase in GFP fluorescence is observed.

To demonstrate the generality of our approach, we generated a MiRAR for another miRNA, miR-122. miR-122 is associated with both Hepatitis B and C infections [42,43,44] and hepatic cancer [45,46,47,48,49]. We used an miR-122-responsive CBEB 3′-UTR in generating this MiRAR contruct. The CBEB 3′-UTR contains only two miR-122 binding sites [38], compared to the five binding sites of let-7 in our KRas-derived MiRAR. Consistent with a reduced number of miRNA binding sites, we observed that a significant change in GFP fluorescence was only observed upon an increase to 100 nM of transfected miR-122 compared to 80 pM in the MiRAR-let-7. The MiRAR-miR-122 reporter is thus approximately 1000-fold less sensitive to changes in miRNA concentration. Thus, our data indicate that the sensitivity of the MiRAR can be tuned depending on the concentration range of miRNA of interest. In the future, we envision that changes in sensitivity or adaptation to other miRNA species can be achieved by either fusing the 3′-UTR of a miRNA-regulated gene to *gfp*, or by mutating the binding sites of e.g., miR-122 or let-7 in the existing constructs to another seed sequence.

In summary, we present a versatile reporter system that is adaptable to different miRNAs and is scalable in terms of sensitivity. Changes in miRNA metabolism in response to extracellular stimuli or over the lifetime of a cell can be monitored in a time resolved manner at the single-cell level without further interfering with the cellular environment. This tool will allow unprecedented insight into miRNA metabolism and biology. Future efforts will include the generation of stable cell lines containing MiRARs directed at certain miRNAs, and adaptation of the current constructs to other target miRNAs. Compared to luciferase-based reporters, our GFP-based reporter circumvents use of chemicals (luciferin) as an additional screening step and may provide a useful tool for high-throughput screening of small molecule chemotherapeutics that are effective in altering miRNA content or activity.

## Supplementary Materials

The following are available online at http://www.mdpi.com/2073-4425/9/6/305/s1, File S1: Reporter gene constructs.
