# Supplementary material for: MiRAR—miRNA Activity Reporter for Living Cells
Source: Genes (Basel). 2018 Jun 19;9(6):305. doi: 10.3390/genes9060305 (PMC6027049; doi:10.3390/genes9060305)
Supplement: Supplementary file 1 [file genes-09-00305-s001.pdf]

Reporter gene constructs:

1. KRAS 3′-UTR into GFP-pcDNA3.1

5′AAGCTTATGGGCAGCAGCCATCATCATCATCATCACAGCAGCGGCCTGGTGCCGCGCGGCAGC
CGTACGATGAGCAAAGGAGAAGAACTTTTCACTGGAGTTGTCCCAATTCTTGTTGAATTAGA
TGGTGATGTTAATGGGCACAAATTTTCTGTCCGTGGAGAGGGTGAAGGTGATGCTACAAAC
GGAAAACTCACCCTTAAATTTATTTGCACTACTGGAAAACTACCTGTTCCATGGCCAACACTT
GTCACTACTCTGACCTATGGTGTTCAATGCTTTTCCCGTTATCCCGATCACATGAAACGGCAT
GACTTTTTCAAGAGTGCCATGCCCGAAGGTTATGTACAGGAACGCACTATATCTTTCAAAGA
TGACGGGACCTACAAGACGCGTGCTGAAGTCAAGTTTGAAGGTGATACCCTTGTTAATCGTA
TCGAGTTAAAAGGTATTGATTTTAAAGAAGATGGAAACATTCTCGGACACAAACTCGAGTAC
AACTTTAACTCACACAATGTATACATCACGGCAGACAAACAAAAGAATGGAATCAAAGCTAA
CTTCAAAATTCGCCACAACGTTGAAGATGGTTCCGTTCAACTAGCAGACCATTATCAACAAA
ATACTCCAATTGGCGATGGCCCTGTCCTTTTACCAGACAACCATTACCTGTCGACACAATCTG
TCCTTTCGAAAGATCCCAACGAAAAGCGTGACCACATGGTCCTTCTTGAGTTTGTAACTGCT
GCTGGGATTACACATGGCATGGATGAGCTCTACAAAGTTAACGGTGGCGGTGGCTCTGGAG
GTGGTGGGTCCGGTACCTAACGGACGCGTGGGCGGCAGCGCTGTGGCGGCGGCTGAGACGGC
AGGGGAAGGCGGCGGCGGCTCGGCCCGGAGTCCCGCTCCCGCGCCATTTCGGACCCGGAGCGA
GCGCGGCGCGGGCCTGAAGGCGGCGGCGGGAGCCTGAGGCGCGGCGGCTCCGCGGCGCGGAGA
GAGGCCTGCTGAAAATGACTGAGTATAAACTTGTGGTGGTTGGAGCTGGTGGCGTAGGCAAGAG
CGCCTTGACGATACAGCTAATTCAGAATCACTTTGTGGATGAGTACGACCCTACGATAGAGGACT
CCTACAGGAAACAAGTAGTAATTGATGGAGAAACCTGTCTCTTGGATATTCTCGACACAGCAGG
TCAAGAGGAGTACAGTGCAATGAGGGACCAGTACATGAGAACTGGGGAGGGCTTTCTTTGTGTA
TTTGCCATAAATAATACTAAATCATTTGAAGATATTCACCATTATAGAGAACAAATTAAAAGAGT
AAAGGACTCTGAAGATGTGCCTATGGTCCTGGTAGGGAATAAGTGTGATTTGCCTTCTAGAACAG
TAGACACGAAACAGGCTCAGGAGTTAGCAAGGAGTTACGGGATTCCGTTCATTGAGACCTCAGC
AAAGACAAGACAGGGTGTTGACGATGCCTTCTATACATTAGTCCGAGAAATTCGAAAACATAAA
GAAAAGATGAGCAAAGATGGGAAGAAGAAGAAGAAGAAGTCAAGGACAAGGTGTACAGTT
ATGTGAATACTTTGTACTCTTTCTTAAGGCACACTTAAGTAAAAGTGTGATTTTTGTACATTACAC
TAAATTATTAGCATTTGTTTTAGCATTACCTAATCTTTTTTTTTTCTTCTGTTCGTGCAAACTGTCAG
CTTTTATCTCAAATGCTTATTTTAAAAGAACAGTGGAAACCTTCTTTTTTCTAAGTGCCAGTATTCC
CTGGGTTTTGGACTTAAACTAGCAATGCCTGTGGAAGAGACTAAAGACCTGAGACTCTGTCTTGG
GATTTGGTGCATGCAGTTGATTCCTTGCTAGTTCTCTTACCAACTGTGAACACTGATGGAAGCAG
GATAATGAAGCTTCCGGACCATCCCTGCTCTGTGTCCATCTACTCATCCAATGGAGTCATTAGCA
GTCAATCGCAGCTTCACTGGACACTGAGGGGTCACAGACTTAGGCTCCCTTTGAGTCACGTCCAG
CGTGTCCTAGACTTTATCATCTTTCAGAGGCGTAGGCAGACTGTTCACAAAGGCTTTCTCTAGCTT
TCCACTGCAATTAATCTTGGTCACTCCCTCAAATAGTATATTTTTTCTAGAAAAGGGGAAAAATG
GAAAAAAAAAAAAAAGGCAATGGAAAATGTTGAAATCCATTCAGTTTCCATGTTAGCTAAATTAC
TGTAAGATTCCTATAATAGCTTTTCCTGGTAAGGCAGACCCAGTATGAAATAGTAATAACCATTT
GGGCTATATTTACATGCTACTAAATTTTTGTAATAATTCAAACAACTTTAGCATATATAAAAAGTT
CTCATAAGAATTAAGTACAGGATCCGACGGATCGGGAGATCTC

HIS-tag GFP, Linker, 3′-UTR, Let-7 binding sites (corresponds to Kumar et al., 2007; miRTarBase, 2018)

2. CPEB 3′-UTR into GFP-pcDNA3.1

5′AAGCTTATGGGCAGCAGCCATCATCATCATCATCACAGCAGCGGCCTGGTGCCGCGCGGCAGC
CGTACGATGAGCAAAGGAGAAGAACTTTTCACTGGAGTTGTCCCAATTCTTGTTGAATTAGA
TGGTGATGTTAATGGGCACAAATTTTCTGTCCGTGGAGAGGGTGAAGGTGATGCTACAAAC
GGAAAACTCACCCTTAAATTTATTTGCACTACTGGAAAACTACCTGTTCCATGGCCAACACTT
GTCACTACTCTGACCTATGGTGTTCAATGCTTTTCCCGTTATCCCGATCACATGAAACGGCAT
GACTTTTTCAAGAGTGCCATGCCCGAAGGTTATGTACAGGAACGCACTATATCTTTCAAAGA
TGACGGGACCTACAAGACGCGTGCTGAAGTCAAGTTTGAAGGTGATACCCTTGTTAATCGTA
TCGAGTTAAAAGGTATTGATTTTAAAGAAGATGGAAACATTCTCGGACACAAACTCGAGTAC
AACTTTAACTCACACAATGTATACATCACGGCAGACAAACAAAAGAATGGAATCAAAGCTAA
CTTCAAAATTCGCCACAACGTTGAAGATGGTTCCGTTCAACTAGCAGACCATTATCAACAAA
ATACTCCAATTGGCGATGGCCCTGTCCTTTTACCAGACAACCATTACCTGTCGACACAATCTG
TCCTTTCGAAAGATCCCAACGAAAAGCGTGACCACATGGTCCTTCTTGAGTTTGTAACTGCT
GCTGGGATTACACATGGCATGGATGAGCTCTACAAAGTTAACGGTGGCGGTGGCTCTGGAG
GTGGTGGGTCCGGTACCTAAAGGAGCTGGCCTTGCCCAGTGGCCTGTGGCGCCCAAAGCTGGC
AGGTCAGGCAAGCAGCCTGCACCACCCTGCCACTGGCGACCAGGGAGCTGGCTTCCCAAGGACA
AGGGAAAATTGTAGTCACCTTTGCACTTGCTGAATCTGTCTTTGTTTCTGCACTAATTAATGCACA
TTGAGTTTTGTCAGGTTTTGTTTTCAGGGGGTGTACCAAGGGCAAGGACCCTCTGGCTTACCCTCC
AAGCGACTCTGTAGTTTTCCCAGATTTTAGTTCCTCATTTTGCAGATGAAAAGCGGGGAAAAAAA
AAAAAAAAAAAAAAAACTACGTGTCCAGAAGGTATTGAGGTATTGACACGGATGCCTACACCTA
GGTTTATTTATTAAAAGCGCTTTTTTACATTCCTTGCAATACTGATGGTGATGATGCGCAGGTCTC
ATTGGTTTCATTCTTGCAGTTGCCATACAGTGCCTTTCCATTTATTTAACCCCCACCTGAACGGCAT
AAACTGAGTGTTCAGCTGGTGTTTTTTACTGTAAACAATAAGGAGACTTTGCTCTTCATTTAAACC
AAAATCATATTTCATATTTTACGCTCGAGGGTTTTTACCGGTTCCTTTTTACACTCCTTAAAACAGT
TTTTAAGTCGTTTGGAACAAGATATTTTTTCTTTCCTGGCAGCTTTTAACATTATAGCAAATTTGTG
TCTGGGGGACTGCTGGTCACTGTTTCTCACAGTTGCAAATCAAGGCATTTGCAACCAAGAAAAAA
AAAATTTTTTGTTTTATTTGAAACTGGACCGGATAAACGGTGTTTGAGCGGCTGCTGTATATAGTT
TTAAATGGTTTATTGCACCTCCTTAAGTTGCACTTATGTGGGGGGGTGGGGTTGATAGAAGTTTTT
AATCACAAAGTCACAGGACTTTTTTCTTTTGTAACTGAGCTAAAAAGGGCTGCTTTTCGGTGGGG
GCAGATGAAGGCTCACAGGAGCCCTTTCTCTTAGAGGGGGCAAGTACCCTTCCCTTATATCTTTA
ATTTGAGGAATGTATGAGATAACAGTTGCAGTTGACTGAAATGCCACTGGAATTTGAAAACTTGA
CTTTTTTTTCTTTCCTTTTTTTTTTTTTTTTTTTTTTTAAAAAGAAACAAAAAACCCACAAAAACTAC
TTGCCCCTCCTAGGGAAGCTGTGTGCCAAAGAACCAGTGTCATAACCCCTCCCTCCTGCTGAGCT
GACGTTGCATTGTTGCATATCCCAGCTACTCTGTGGGTTTTGTGAAGCTGTGTGTGAAGTCTCTAC
CTCATTGTAGTATATGCAGGCAAGACTGCACTCTCCTACAGATGTGTGGAGTAAGCTGTGGTGTA
GTTTTTTTGGCACATAATAAACACGTTGCAGCAG**GGATCC**GACGGATCGGGAGATCTCCCGATCC
CCTATGGTCG

HIS-tag GFP, Linker, 3′-UTR, miR-122 binding sites (corresponds to Burns et al. 2011)

GGTACC – *Kpn*I

GGATCC – *Bam*HI
